# Mixing Acid Salts and Layered Double Hydroxides in Nanoscale under Solid Condition

**DOI:** 10.3390/pharmaceutics6030436

**Published:** 2014-07-30

**Authors:** Hirokazu Nakayama, Aki Hayashi

**Affiliations:** Department of Functional Molecular Chemistry, Kobe Pharmaceutical University, Kobe 658-8558, Japan; E-Mail: ahayashi@kobepharma-u.ac.jp

**Keywords:** intercalation, carrier, layered double hydroxide, hydrotalcite

## Abstract

The immobilization of potassium sorbate, potassium aspartate and sorbic acid in layered double hydroxide under solid condition was examined. By simply mixing two solids, immobilization of sorbate and aspartate in the interlayer space of nitrate-type layered double hydroxide, so called intercalation reaction, was achieved, and the uptakes, that is, the amount of immobilized salts and the interlayer distances of intercalation compounds were almost the same as those obtained in aqueous solution. However, no intercalation was achieved for sorbic acid. Although intercalation of sorbate and aspartate into chloride-type layered double hydroxide was possible, the uptakes for these intercalation compounds were lower than those obtained using nitrate-type layered double hydroxide. The intercalation under solid condition could be achieved to the same extent as for ion-exchange reaction in aqueous solution, and the reactivity was similar to that observed in aqueous solution. This method will enable the encapsulation of acidic drug in layered double hydroxide as nano level simply by mixing both solids.

## 1. Introduction

Recently, the idea of molecular capsules, including polymers, liposomes and dendrimers, has attracted much attention [1] with a view to the development of drug delivery systems; layered ceramic compounds and porous compounds have been considered as candidates for such capsules. Hydrotalcite-like compounds with a general formula M_1−*x*_^2+^M*_x_*^3+^(OH)_2_(A^*n*−^)*_x/n_*·*y*H_2_O, where M^2+^ and M^3+^ are di- and trivalent metals, respectively, and A^*n*−^ is an interlayer anion, are generally called layered double hydroxides (LDHs) or hydrotalcite. LDHs have been used as antacids in pharmaceutical applications [2] and adsorbents for phosphate [3,4]. Recently, intercalation—the incorporation of organic anions into the interlayer space by an ion-exchange reaction—has been used for the synthesis of drug–inorganic hybrid materials for use as molecular capsules [5,6,7,8,9].

Until now, intercalation reaction has normally been performed in aqueous solution by ion exchange, reconstruction, or co-precipitation method [10]. However, if the intercalation under solid condition would be possible, it is economical and quick without limitation of solvent. Therefore, this would facilitate the synthesis of drug–inorganic hybrid materials with no waste, thus extending their applicability. The formation of inorganic–organic hybrid under dry condition has been reported for montmorillonite with amines [11,12]. Intercalation of volatile 8-hydroquinoline into smectite and *in situ* complexation with Zn^2+^ in the interlayer space under solid was also reported [13]. However, has been few pioneering reports for LDH [14,15], because layer charge of the LDH host is considerably high compared with those of the above clays, and strong electrostatic interaction between positive layer and interlayer anion will halt this process under solid. However, we recently found that the intercalation of sodium valproate into nitrate-type LDH could be achieved by mixing sodium valproate and LDH, both in powder form, for a few minutes [16].

Drugs are normally mixed with various types of additives and binders, and are dispersed in them in tablet. Normally, drugs are dispersed as microscopic level, and the tablet is still a mixture of drug and additives. If our methodology can be applied to various types of drugs, then drugs are dispersed in the nanometer scale, and are covered by layered double hydroxide in the molecular scale simply by mixing drugs and LDH solids. This would enable the enhanced stability of drug. In the case of sodium valproate, as mentioned above, complete protection of humidity is possible by simply mixing the solids. Therefore, this methodology would contain potential application. In this report, we extend this method to various types of compound to clarify its applicability and limitation.

## 2. Materials and Methods

### 2.1. Materials

Nitrate-type LDH, Mg_0.66_Al_0.33_(OH)_2.00_(NO_3_)_0.27_(CO_3_)_0.02_·0.20H_2_O (abbreviated as LDH(NO_3_)), was prepared by a co-precipitation method [17]. Chloride-type LDH, Mg_0.69_Al_0.31_(OH)_2.03_Cl_0.26_ (CO_3_)_0.01_·0.48H_2_O (abbreviated as LDH(Cl)), and carbonate-type LDH, Mg_0.75_Al_0.25_(OH)_2.00_ (CO_3_)_0.125_·0.52H_2_O (abbreviated as LDH(CO_3_)), were purchased from Tomita Chemical Co., Ltd. (Tokushima, Japan). Other chemicals of research grade—sodium valproate (VPA-Na), potassium sorbate (SA-K), potassium aspartate (Asp-K), and sorbic acid (SA)—were purchased from Wako Chemical Co., Ltd. (Osaka, Japan).

### 2.2. Preparation of Intercalation Compounds

Powdered LDH(NO_3_), LDH(Cl) or LDH(CO_3_) (0.1 g) was ground with 0.01–0.1 g of VPA-Na, SA-K, Asp-K or SA powder in an agate mortar for 5 min at room temperature, and the resulting solid was dried under vacuum for 3 h at room temperature. For the intercalation reaction in aqueous solution, 0.1 g of LDH(NO_3_), LDH(Cl) or LDH(CO_3_) was stirred in an aqueous solution of VPA-Na, SA-K, Asp-K or SA (1–90 mmol/L) for 30 min.

### 2.3. Characterization

The obtained solid was characterized by X-ray diffraction (XRD), solid-state NMR and FT-IR. The uptake of VPA-Na, SA-K, Asp-K or SA was determined by elemental analysis. Powder XRD was employed to monitor the intercalation compound, using a Rigaku Denki Rint 2000 diffractometer (Rigaku Coporation, Tokyo, Japan) with Ni-filtered Cu*K*α radiation. The interlayer distances (*d*) were calculated from Bragg’s equation, 2*d* sin = *n*, using the lowest angle peak from the XRD patterns. Solid-state ^13^C CP/MAS NMR spectra of the intercalation compounds were recorded using a Varian NMR system AS500 spectrometer (Agilent Technologies, Inc., Santa Clara, CA, USA) operating at 125.7 MHz, with a MAS rate of 20 kHz and a recycle delay of 5 s with the accumulation of 100–12,000 scans. FT-IR spectra were measured by Thermo Electron 200 spectrometer (Thermo Electron, Madison, WI, USA) with the resolution of 4 cm^−1^. Elemental analysis of carbon in the intercalation compound was performed using a Sumigraph NC-80 elemental analyzer (Sumika Chemical Analysis Service, Ltd., Osaka, Japan).

## 3. Results and Discussion

### 3.1. Intercalation with LDH(NO_3_)

In the previous letter, we reported that intercalation of sodium valproate into nitrate-type LDH could be achieved by mixing both powder for a few minutes [16]. Because sodium valproate (VPA-Na) is very hygroscopic, it was suspected that the presence of water might assist the solid-solid intercalation reaction, and this method can be applied only for hygroscopic compounds. In order to examine the applicability of solid-state intercalation reactions, the intercalation was carried out using three different types of compounds: potassium sorbate (SA-K), potassium aspartate (Asp-K), and sorbic acid (SA). In order to check whether hygroscopic property is essential for solid-solid intercalation, non-hygroscopic SA-K was selected. Asp-K is a zwitterion, which is sometimes difficult to intercalate even in aqueous solution. The control of pH is important in aqueous intercalation reaction, but it is impossible to control pH in a solid-solid intercalation reaction; thus, we compared the reactivity of SA-K and SA directly. Although nitrate type of LDH is very reactive for the intercalation reaction, residual NO_3_^−^ may inhibit the pharmaceutical application. We then examined the reactivity of chloride type of LDH.

Because the addition of tiny aliquots of water has been found to accelerate the intercalation reaction [16], 30 L of H_2_O was added before grinding. This amount of H_2_O is extremely small compared to roughly 0.1 g of solid, so it was impossible to detect with the eyes during grinding, but it was very effective in accelerating the reaction.

**Figure 1:**
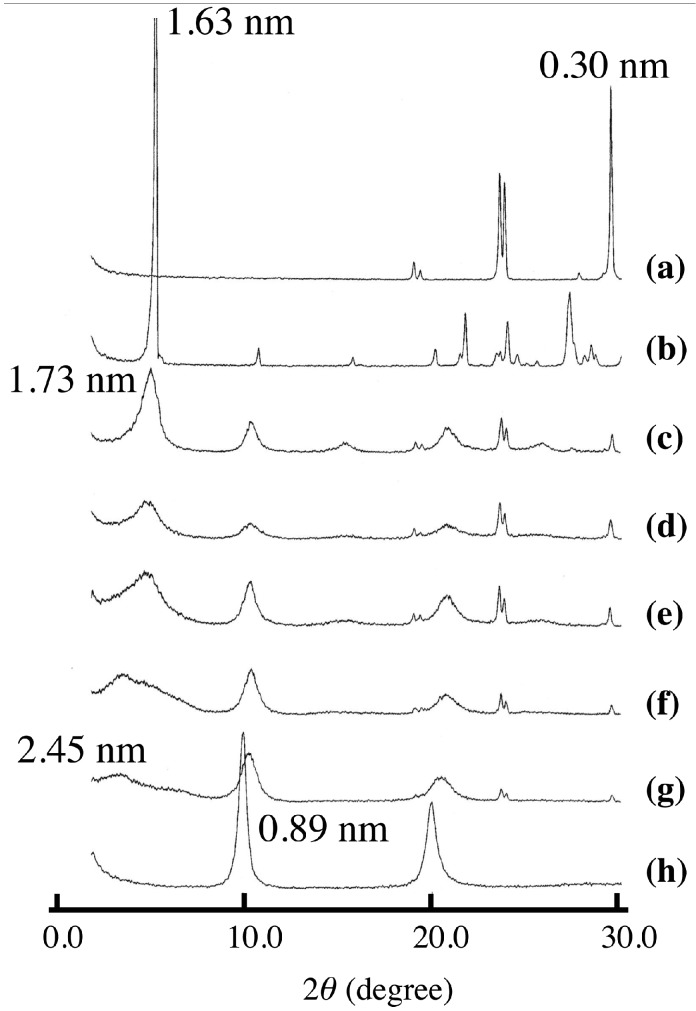
X-ray diffraction (XRD) patterns of (**a**) KNO_3_; (**b**) potassium sorbate (SA-K); and (**c**–**g**) layered double hydroxides (LDH)(NO_3_) ground with various amounts of SA-K for 5 min at room temperature (recorded before the samples were washed with water). (**c**) 4.7; (**d**) 3.3; (**e**) 2.8; (**f**) 2.3; (**g**) 1.3 mmol per 1 g LDH(NO_3_). XRD pattern of (**h**) host LDH(NO_3_) is shown for comparison.

**Figure 2:**
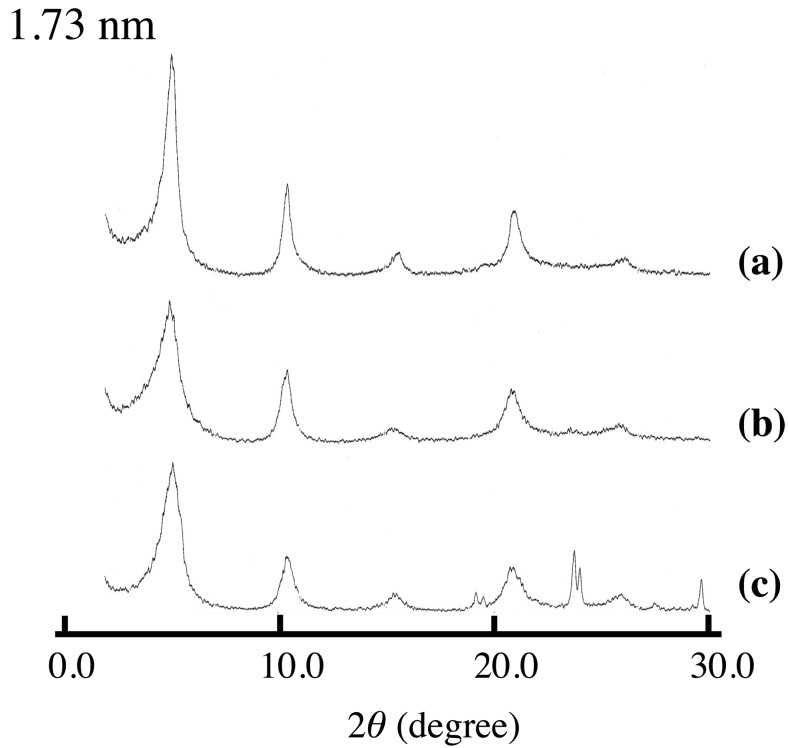
XRD patterns of (**a**) SA-K intercalated LDH(NO_3_) synthesized in aqueous solution, and LDH(NO_3_) ground with 1.3 mmol per 1 g LDH(NO_3_) for 5 min at room temperature (**b**) after and (**c**) before washing with water.

Figure 1 shows the XRD patterns of LDH(NO_3_) ground with various amounts of SA-K. Two new diffraction peaks with interlayer distances of 1.73 and 2.45 nm were observed together with the peaks of LDH(NO_3_) and SA-K, suggesting that the intercalation of sorbate into the interlayer space of LDH through a solid-solid reaction was successful. On increasing the added amount of SA-K, the new diffraction peak shifted, grew in size and became sharper as shown in Table 1. A decrease in the peak intensities of LDH(NO_3_) was observed at the same time. Furthermore, new diffraction peaks at 2θ = 24° and 29° appeared after the reaction. These were thought to be due to KNO_3_, possibly due to a reaction in the solid mixture between nitrate ions in the interlayer space of LDH(NO_3_) and potassium ions of SA-K. After the solid product was washed with water, the diffraction peaks due to SA-K and KNO_3_ disappeared completely and the new peak with an interlayer distance of 1.73–2.45 nm remained, which suggested that the intercalation compound had been separated (Figure 2). From XRD data there observed two different intercalation compounds with the interlayer space of 2.45 and 1.73 nm. The intercalation compound with the interlayer space of 1.73 nm was observed in intercalation reaction in aqueous solution as shown in Figure 2. However, the intercalation compounds with the interlayer space of 2.45 nm could not be observed in the reaction of aqueous solution. Therefore, this phase is peculiar to solid-solid reaction. In intercalation reactions, several phases are sometimes observed at different conditions. However, the intercalation compound with lager interlayer distance is normally observed at the reaction with higher concentration. This is not the case. Moreover, 2.45 nm is almost the same as that of summation of 1.73 and 0.89 nm of nitrate-type LDH itself. Therefore, a second staging phase might be observed at the small addition of SA-K.

**Table 1:** Added amount of SA-K, interlayer distance (*d*), full width at half maximum (FWHM) of XRD and uptake of SA-K using nitrate-LDH in solid-state reaction.

Added Amount of SA-K (mmol/g)	Interlayer Distance (nm)	FWHM	Uptake Amount of SA-K (mmol/g)
4.7	1.73	1.0	2.5
3.3	1.73	1.3	2.1
2.8	1.73	1.5	1.9
2.3	2.45	2.0	1.6
1.3	2.45	2.3	1.0

**Figure 3:**
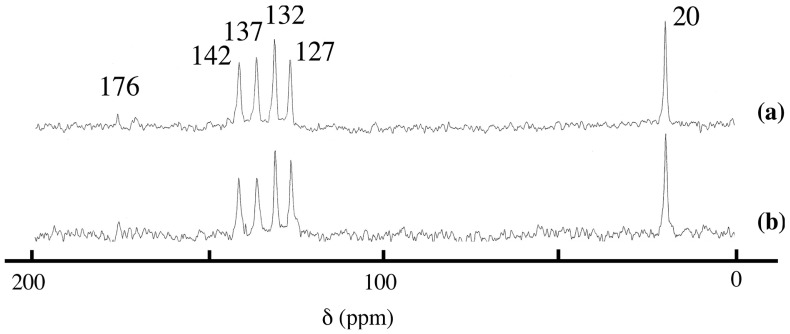
^13^C CP/MAS NMR spectra of sorbate-intercalated LDH obtained (**a**) in a solid-state reaction (sample of Figure 1c) and (**b**) in aqueous solution.

As shown in Figure 3, the ^13^C CP/MAS NMR spectrum of the washed product was almost the same as that of sorbate-intercalated LDH synthesized in aqueous solution, suggesting the presence of sorbate anion in the interlayer space of LDH. The chemical shifts of the intercalation compound were similar to those of SA-K in aqueous solution, with a small shift, which suggested that no decomposition of sorbate took place during grinding. The chemical shift values of the product are different from those of solid SA-K, suggesting no surface adsorption. It was, therefore, concluded that ion exchange had proceeded in the solid state. Although it was impossible for us to detect the existence of water, it was expected to accelerate the migration of anions during grinding. The obtained intercalation compound is almost the same as that synthesized in aqueous solution [14,15].

**Figure 4:**
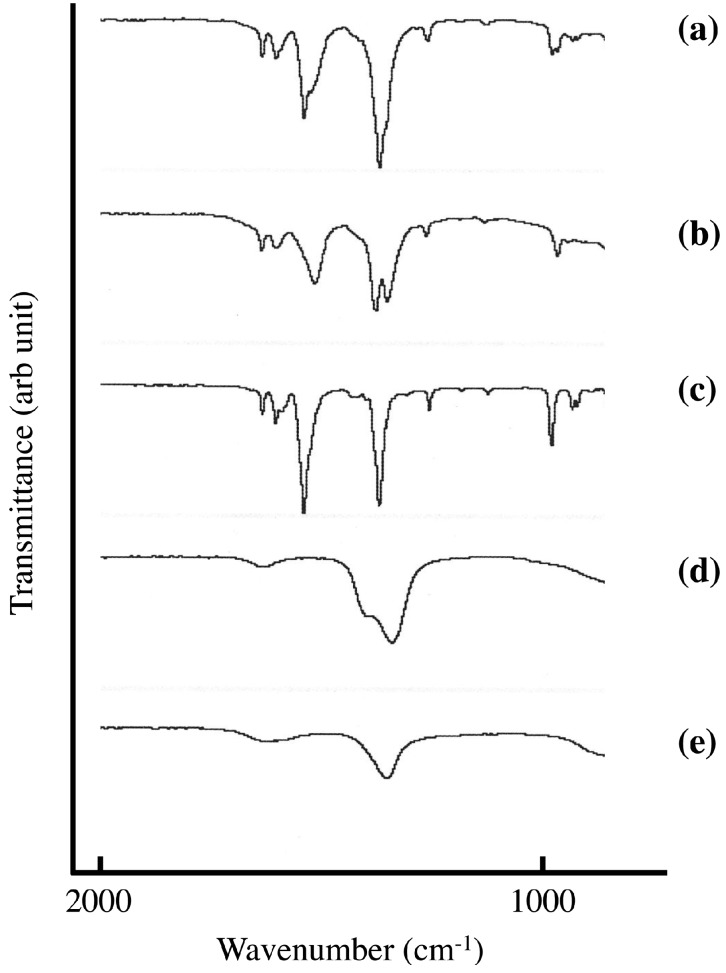
FT-IR spectra of (**a**) LDH(NO_3_) ground with 1.3 mmol of SA-K per 1 g LDH(NO_3_) for 5 min at room temperature; and (**b**) SA-K intercalated LDH(NO_3_) synthesized in aqueous solution; FT-IR spectra of (**c**) SA-K; (**d**) LDH(NO_3_); and (**e**) LDH(CO_3_) were shown for comparison.

Figure 4 shows FT-IR spectra of sorbate-intercalated LDH synthesized in the solid-solid reaction and in aqueous solution. The occlusion of carbonate anion through CO_2_ in air is a serious problem in the intercalation reaction in aqueous solution. Because the affinity of carbonate anion in the LDH is very high, it is very difficult to remove it. The adsorption around 1300 cm^−1^ shows doublet in sorbate intercalated LDH synthesized in aqueous solution. The adsorption at 1398 cm^−1^ would be due to sorbate (Figure 4c) and the other would be due to carbonate in LDH (Figure 4e). On the other hand LDH(NO_3_) ground with 1.3 mmol of SA-K per 1 g LDH(NO_3_) shows singlet at 1388 cm^−1^ around 1300 cm^−1^, and no adsorption due to carbonate anion could not be observed. The removal of carbonate anion would be attained in the solid-solid reaction. In the solid-solid reaction, the reaction time is 5 min, which is extraordinary short with the reaction in aqueous solution. The first step of occlusion of carbonate anion is dissolution of gaseous CO_2_ in aqueous solution. This is not the case in solid-solid reaction because there is no solution. Therefore, this is very big advantage compared with the reaction in aqueous solution.

**Figure 5:**
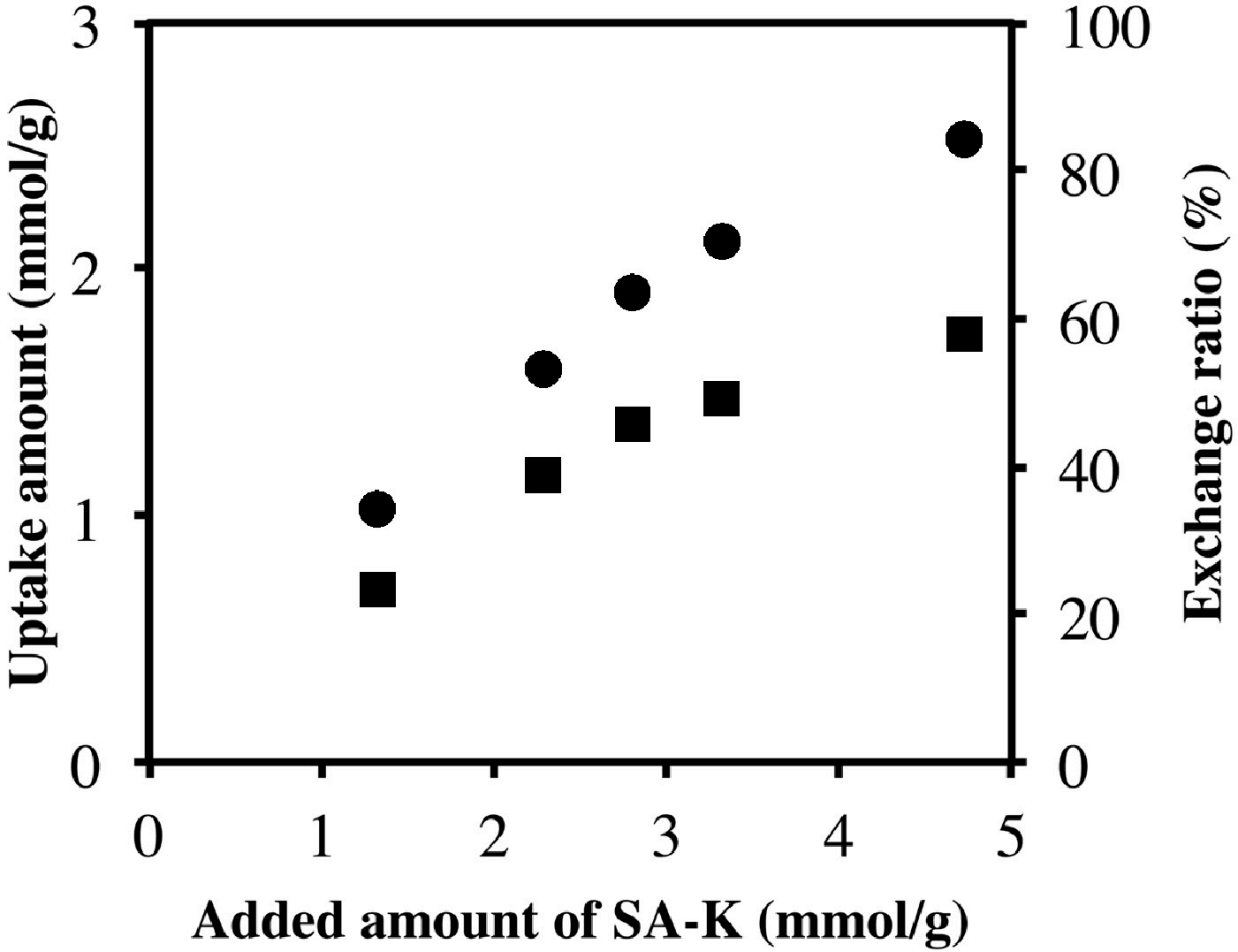
Uptakes (●) and exchange ratios (■) of sorbate for various amounts of SA-K added.

Figure 5 shows the uptake amount (exchange amount) of sorbate in the intercalation compound at various amounts of SA-K added. When the amount of SA-K was increased, the uptake of sorbate was also found to increase; however, the exchange ratio, which corresponds to (uptake amount/ion exchange capacity of LDH) × 100, reached a maximum of 60% at 4.7 mmol of SA-K added per 1 g of LDH. This fact is consistent with the presence of peaks due to unreacted LDH(NO_3_) in Figure 1c. The amount of SA-K added in the solid-solid intercalation reaction corresponds to the concentration in the aqueous reaction. The concentration is the driving force of ion exchange, and uptake is saturated at higher concentrations. Therefore, Figure 5 supports an ion-exchange mechanism for solid-solid intercalation. Although 4.7 mmol of SA-K represents 1.2 times the ion exchange capacity of 0.1 g LDH(NO_3_), complete exchange was not achieved. As shown in Table 2, this is in contrast with the case of VPA-Na, in which complete solid-solid intercalation was achieved [16].

**Table 2:** Interlayer distance (*d*) and uptake of sodium valproate (VPA)-Na, SA-K, and potassium aspartate (Asp-K) using nitrate-LDH in solid-state reaction and in aqueous solution.

Compound	Interlayer Distance (nm)		Uptake (mmol/g LDH)	
	Solid	Solution	Solid	Solution
VPA-Na	1.8	1.8	3.4	3.8
SA-K	1.7 2.5	1.7 2.5	2.5	2.4
Asp-K	1.2	1.2	1.8	2.1

Intercalation of SA was not possible in solid-state reaction. In fact the dissolution of LDH, which is composed of hydroxides, by the acid, and increase of pH induces the formation of sorbate anions and allows intercalation to progress in aqueous solution, but this was not possible in the solid state. This result also supports the ion exchange mechanism of solid-solid intercalation.

**Figure 6:**
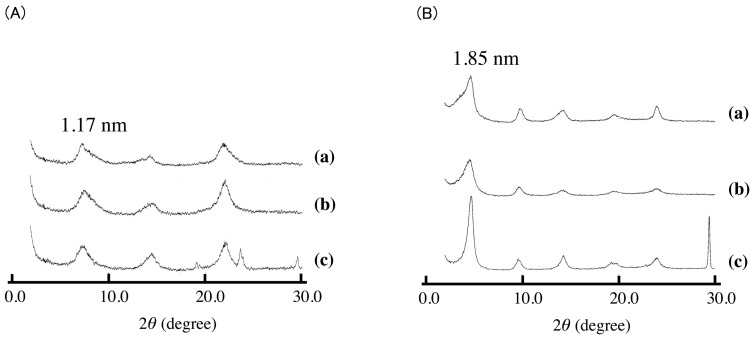
XRD patterns of (**A**) ASP-K intercalated LDH(NO_3_) and (**B**) VPA-Na intercalated LDH(NO_3_). (**a**) is the XRD pattern of sample synthesized in aqueous solution, and (**b**) and (**c**) are XRD patterns of samples synthesized under solid condition. (**b**) and (**c**) are XRD patterns after and before washing sample with water.

Similar experiments were performed for Asp-K. In the case of Asp-K, an increase in the interlayer distance was observed as shown in Figure 6. Together with the peaks due to the intercalation compound, the peaks due to KNO_3_ were observed at the same time. The removal of the peaks of KNO_3_ was observed after washing with water. The intercalation under solid condition was concluded to be possible for acid salts such as VPA-Na, SA-K, and Asp-K. The interlayer distances of these intercalation compounds were almost the same as those of the equivalent compounds obtained in aqueous solution; it was therefore concluded that solid-state intercalation resulted in the same intercalation compounds as aqueous intercalation. Therefore, the mechanism of the intercalation is almost the same as VPA-Na (Figure 6B) and SA-K. As shown in Table 2, the uptakes for solid-state intercalation were also the same as those achieved in aqueous solution, indicating that the reactivity was almost the same as for the ion-exchange reaction in solution. In contrast, with the case of VPA-Na, in which complete exchange was achieved, the exchange ratio was a maximum of 60% and 42% for SA-K and Asp-K. In particular, in the case of Asp-K, the exchange ratio is almost half of VPA-Na. Asp-K is zwitterion, and the existence of positive charge near the carboxylic acid lowers the reactivity of intercalation reaction in aqueous solution. This is the case even in the solid-solid reaction.

Therefore, hygroscopic property is not essential for solid-solid intercalation reaction by adding a very small amount of water. The reactivity of solid-solid intercalation is similar to that in aqueous solution.

### 3.2. Intercalation with LDH(Cl)

Although nitrate type of LDH is very reactive for intercalation reaction, the existence of residual NO_3_^−^ may limit the pharmaceutical application. So we then examined the reaction using other types of LDH, that is, carbonate and chloride type of LDH. It was found that solid-solid intercalation proceeded by an ion-exchange mechanism, and the reactivity of a guest compound in the solid-state reaction was almost the same as that in aqueous solution. Because the anion of the host LDH shows a strong influence in the intercalation reaction in aqueous solution [18], we decided to examine the reactivity of the host LDH using LDH(Cl) and LDH(CO_3_). In the case of LDH(CO_3_), no intercalation reaction occurred for VPA-Na, SA-K, Asp-K and SA. It is well known that the carbonate in LDH(CO_3_) is one of most stable anions in the interlayer space of LDH [18]. Therefore, it is hard to perform an ion-exchange intercalation reaction with LDH(CO_3_) in aqueous solution, and the same tendency was found for solid-solid intercalation. This is thought to be due to a strong coulomb interaction between the carbonate anions and the positively charged LDH layer.

LDH(Cl) was found to behave differently from LDH(NO_3_). Table 3 summarizes the uptake of guest compounds in the intercalation reactions. For LDH(Cl), solid state intercalation proceeded, but the reactivity was complex. In the case of VPA-Na, uptake was small. The peak due to the intercalation compound could not be observed, and a large peak due to unreacted LDH(Cl) remained. The presence of KCl in the XRD pattern verified that solid-solid intercalation had taken place. The absence of a peak corresponding to the intercalation compound was thought to be due to a low exchange ratio. It was thought that the broadness of the peak of the intercalation compound prevented its observation. The same tendency was observed for Asp-K. The situation was the same as that of SA-K. The uptakes of the guest compounds in solution were low compared with those for LDH(NO_3_). Therefore, a lowering of reactivity for both the solid-state reaction and the reaction in solution was verified, with almost the same uptakes observed in the two types of reaction.

**Table 3:** Uptake of VPA-Na, SA-K, and Asp-K in LDH(Cl) in solid-state reaction and in solution.

Compound	Uptake (mmol/g LDH)	
	Solid	Solution
VPA-Na	0.6	0.4
SA-K	1.1	0.5
Asp-K	0.8	1.2

It was concluded that solid intercalation using LDH(Cl) was possible, and that the reactivity in the solid state was almost the same as that in aqueous solution; however, it was lower than that of LDH(NO_3_). This was due to the low activity of LDH(Cl) compared to LDH(NO_3_).

## 4. Conclusions

The intercalation of sorbate and aspartate into nitrate-type LDH was achieved, and the uptakes and interlayer distances of the intercalation compounds obtained in the solid-state reaction were the same as those of the equivalent compounds obtained in aqueous solution. Although the intercalation of sorbate and aspartate into chloride-type LDH was possible, the uptake was low compared with the intercalation compounds obtained using nitrate-type LDH. Solid-solid intercalation appears to be possible to the same level as that achieved through ion-exchange in aqueous solution, with similar reactivity.
